# The role of payment and financing in achieving health equity

**DOI:** 10.1111/1475-6773.14219

**Published:** 2023-11-28

**Authors:** Brede H. Eschliman, Hongmai H. Pham, Amol S. Navathe, Karen M. Dale, Julian Harris

**Affiliations:** ^1^ Medicare Drug Rebate and Negotiations Group Centers for Medicare and Medicaid Services Windsor Mill Maryland USA; ^2^ Institute for Exceptional Care Washington DC USA; ^3^ Department of Medical Ethics and Health Policy, Perelman School of Medicine University of Pennsylvania Philadelphia Pennsylvania USA; ^4^ Corporal Michael J. Cresencz VA Medical Center Philadelphia Pennsylvania USA; ^5^ AmeriHealth Caritas DC Washington DC USA; ^6^ Deerfield Management New York New York USA; ^7^ ConcertoCare New York New York USA

**Keywords:** health expenditures, healthcare policy, healthcare reform, hospital costs, National Health Policy

## Abstract

**Objective:**

The aim was to identify healthcare payment and financing reforms to promote health equity and ways that the Agency for Healthcare Research and Quality (AHRQ) may promote those reforms.

**Data Sources and Study Setting:**

AHRQ convened a payment and financing workgroup–the authors of this paper–as part of its Health Equity Summit held in July 2022. This workgroup drew from its collective experience with healthcare payment and financing reform, as well as feedback from participants in a session at the Health Equity Summit, to identify the evidence base and promising paths for reforms to promote health equity.

**Study Design:**

The payment and financing workgroup developed an outline of reforms to promote health equity, presented the outline to participants in the payment and financing session of the July 2022 AHRQ Health Equity Summit, and integrated feedback from the participants.

**Data Collection/Extraction Methods:**

This paper did not require novel data collection; the authors collected the data from the existing evidence base.

**Principal Findings:**

The paper outlines root causes of health inequity and corresponding potential reforms in five domains: (1) the differential distribution of resources between healthcare providers serving different communities, (2) scarcity of financing for populations most in need, (3) lack of integration/accountability, (4) patient cost barriers to care, and (5) bias in provider behavior and diagnostic tools.

**Conclusions:**

Additional research is necessary to determine whether the proposed reforms are effective in promoting health equity.

**What is known on this topic**

- Health outcomes are disparate for patient populations of different races, incomes, disability status, and other characteristics.
- Payment and financing design has an effect on health disparities.

**What this study adds**

- We integrate the existing evidence on causes of health disparities and evaluations of previous health payment reforms efforts.
- We recommend promising payment and financing policies to explore further, with a particular emphasis on the role that AHRQ can play as a research and convening entity.

## 1 INTRODUCTION

Healthcare payment and financing have a critical role to play in achieving health equity. The policies and market forces that control the distribution of resources are a source of inequity as currently structured, and changing them would address multiple root causes of health disparities. Acknowledging the importance of payment and financing to health equity, the Agency for Healthcare Research and Quality (AHRQ) created a payment and financing workgroup as part of its Health Equity Summit held in July 2022. The members of that workgroup are the authors of this paper, which explores ways to use financing and payment to promote health equity, with specific recommendations for areas of research or collaboration that AHRQ could pursue.

For purposes of this paper, we define payment as the mechanism for compensating healthcare providers or other service providers for services and goods (e.g., paying a physician for an office visit) and financing as the mechanism for procuring the funding available for payment (e.g., a state legislature passing a Medicaid budget or health insurers charging enrollees premiums).

### 1.1 Background

Research on the impact of different financing and payment approaches on outcomes including access to services, care quality, and total spending has yielded conflicting results. This is not surprising, given the many contextual factors that mediate or confound the effects in real‐life application.^1^ For example, some factors relate to payments such as the generosity of pricing. Others have little to do with the payments themselves (such as patient cost‐sharing, staffing models, local care‐seeking cultures, and monitoring systems to discourage underuse or overuse) yet mediate their impacts. Thus, it is difficult to conclude that any specific payment structure would produce the best outcomes without knowing the local context. Similarly, the evidence on impacts of financing mediated through health is mixed. For example, different studies found that Medicaid managed care results in higher or lower quality and better or worse access to services, depending on the time period, state, populations, quality domains, and services measured.^2^

Data on the impact of individual payment structures may also be challenging to generalize because of externalities. For example, providers are subject to different financing and payment mechanisms from a multitude of public and private payers, which when layered together may mute or amplify each other's effects.

However, there is clearer evidence of improved access to care and outcomes when people gain access to Medicaid coverage and/or home‐and‐community‐based services (HCBS). Because Medicaid enrollees are disproportionately Black/Brown, poor, and disabled relative to the general population, broadening access to these services has clear potential to reduce health inequity.

More recent healthcare payment reform efforts have focused on the transition from volume‐based care (fee‐for‐service claims reimbursement) to “value‐based” care (payment arrangements that aim to reward and incentivize high‐value care) and reductions in total costs of care, rather than on improving health equity. In particular, the Center for Medicare and Medicaid Innovation (CMMI)—where several of us have worked or consulted—has tested more than 50 alternative payment models that aim to lower costs while improving quality of care in the last decade. It has become clear that the value‐based care models that have been tested have not improved health equity, as CMMI itself recently noted in a strategic white paper.^3^ There are several reasons why value‐based payment reforms have not succeeded in promoting health equity: emphasis on savings, focus on financial risk, and lack of financing reform. Each is described in greater detail below.

### 1.2 Emphasis on savings

Many value‐based care programs have defined success as the generation of savings for payers (either commercial or public). This concept is, in fact, central to the statute that established CMMI. To be tested by CMMI, a payment model must be expected to either be cost‐neutral while improving quality of care or generate federal Medicare or Medicaid savings while improving or maintaining quality of care. As a result, payment models that would dedicate additional resources to underserved populations are less likely to be tested. Medicaid and commercial insurers, operating under relatively fixed revenues, have similar disincentives to increase payments for underserved populations.

### 1.3 Focus on financial risk

The concept of “value” has often been operationalized through alternative payment models that emphasize financial risk borne by providers, such as the Health Care Payment Learning and Action Network's (HCP‐LAN) influential taxonomy of value‐based payment approaches that places priority on financial risk.^4^ While financial risk can have positive effects on cost and outcomes, it has also been found to exacerbate inequities in some circumstances by creating disincentives to treat patients with greater needs.^5^ Research has found that accountable care models (ACOs) are less common in geographic areas where a higher percentage of the population is Black, living in poverty, disabled, or has less than a high school education.^6^ Likewise, communities that have a greater share of residents who are dually eligible for Medicare and Medicaid (indicative of low income) are less likely to have hospitals participating in bundled payment models.^7^

### 1.4 Lack of financing reform

The focus on payment has neglected the critical importance of financing reform, which is also necessary to achieving equity as we describe below.

## 2 METHODS

Prior to AHRQ's Health Equity summit in July 2022, a workgroup consisting of the authors of this paper convened to draft an outline of the paper, drawing from our previous research and experience with payment and finance reforms. A broader group of expert stakeholders was invited to attend a session during the summit to provide feedback on that outline. Incorporating that feedback, the authors came to a consensus on themes, priorities, and recommendations and conducted a literature review to inform recommendations. AHRQ provided feedback on the paper prior to submission to HSR. AHRQ's supplement paper describes this process in more detail.

## 3 RESULTS

In part due to historical prioritization of value‐based payment reforms that lacked explicit equity goals, there is limited evidence on which payment and financing reforms would most effectively promote equity. To identify promising models worth further testing, we start by analyzing potential root causes of inequities that are related to payment and financing, then describe payment and financing reforms that could address those root causes.

### 3.1 Identifying root causes

Many of the root causes of health inequity that are related to financing and payment fit within five domains: (1) the differential distribution of resources between providers serving different communities, (2) scarcity of financing for populations most in need, (3) lack of service integration and clear accountability for outcomes, (4) patient cost barriers to care, and (5) bias in provider behavior and diagnostic tools. Table 1 describes each of these domains in greater detail, with examples of each domain.

**TABLE 1:** Root cause domains.

**Differential distribution of resources between providers**
*Description* Structural factors cause differences in the level of resources available to different provider organizations, which treat different patient populations.
*Examples* Different payers pay different rates for the same service, which advantages providers serving patients with higher‐paying insurance and disadvantages those serving patients with lower‐paying or no insurance. Geographical segregation of patients with different insurance types—often correlated with race, income, disability, and other factors—concentrates higher paying and lower paying insurance in different practices/facilities.^8^ Market power is not evenly distributed between all provider types, allowing some provider organizations (e.g., larger health systems in less competitive markets) to have more success in negotiating favorable payment rates.^9^ This consolidation of market power perpetuates revenue differences across facilities/health systems serving different patient populations.
**Scarcity of financing**
*Description* Policy choices limit the availability of resources necessary for optimal health, and social factors cause some populations to have even less access to already‐scarce services.
*Examples* Medicaid is the single largest insurer in the United States,^10^ with approximately 76 million people enrolled and expenditures over $670 billion annually.^11^ Yet Medicaid financing remains insufficient to offer providers payment rates comparable with those under Medicare or commercial insurance, limiting access to care for patients with Medicaid. Variation in Medicaid financing also creates geographic inequities across states, which have different Medicaid budgets and policies.^12^ Some services necessary for optimal health outcomes are scarce due to policy decisions. For example, the majority of states do not provide home‐ and community‐based services (HCBS) to every person who meets the criteria to receive them.^13^ This scarcity drives disparities between populations who rely on those services for optimal health outcomes (in this example, people with disabilities) and populations who do not need those services for optimal health outcomes (people without disabilities). Many scarce services that are necessary for optimal health are not clinical services—for example, early childhood education, housing, or income support for people living in poverty. Social factors such as race, income, disability, and housing status affect which subpopulations have the resources to navigate access to scarce services, creating further disparities within an already disadvantaged population.^14^
**Lack of integration of services and accountability for outcomes**
*Description* No healthcare entity is accountable for every aspect of the health and wellbeing of a population. As a result, no organization/entity is held responsible if the system as a whole fails to achieve health, especially if the failure is related to non‐clinical needs.
*Examples* People with disabilities receive services and supports from Medicaid,^15^ schools,^16^ and other sources. No entity is responsible for ensuring that there is adequate coordination across those services, or that people with disabilities receive all of the services they need. People have different sources of health coverage over the course of their lifetime, which creates a disincentive for payers to make investments in long‐term health outcomes because the health and financial rewards of doing so would likely accrue to a different payer. Medicaid and the Children's Health Insurance Program (CHIP), which cover about 40% of children in the United States, largely do not benefit from the long‐term savings of childhood interventions that they finance, even if the states that sponsor them may realize savings from children who grow into a healthier adult workforce.
**Patient cost barriers to care**
*Description* Different forms of healthcare coverage entail different patient cost‐sharing for the same services, and even minimal patient cost‐sharing is correlated with lower utilization of both high‐ and low‐value services.^17^
*Examples* States that have not expanded Medicaid eligibility prevent people just above the federal poverty level from accessing Medicaid, a form of insurance that offers significant protections from out‐of‐pocket costs. There is geographic inequity in that some residents of states that have expanded Medicaid eligibility have access to Medicaid coverage with low or no cost‐sharing, but residents of non‐expansion states (largely concentrated in the southeastern United States) with comparable incomes must either rely on other forms of insurance that typically have higher cost‐sharing—exchange plans or employer‐sponsored insurance—or forego insurance.^18^
**Bias in provider behavior and diagnostic tools**
*Description* Provider decisions and diagnostic tools apply a different standard of care to patients with different characteristics, with few financial penalties.^19^, ^20^, ^21^
*Examples* There is evidence of bias influencing providers' care decisions. Providers are less likely to prescribe pain medication^22^ or initiate cardiovascular interventions^23^ for non‐white patients than white patients and more likely to misdiagnose^24^ patients with disabilities than patients without disabilities. Diagnostic tools such as pulse oximeters^25^ or the algorithm^26^ for calculating estimated glomerular filtration rate (eGFR, a measure of kidney function)^27^ have been shown to yield inaccurate results for Black patients.

## 4 DISCUSSION

### 4.1 Payment and financing reforms

A number of payment and financing reforms that have potential to address the root causes have been described above. The potential reforms suggested below vary in the degree of effort required to implement them, from expansions of existing programs that could be accomplished under existing administrative authority to significant financing reforms requiring legislation. We focus recommendations on how AHRQ could support these reforms given its distinct levers—particularly related to research and convening—and do not address the broader advocacy and political strategies that would also be necessary to make these reforms a reality.

### 4.2 Strategies to address differential distribution of resources between providers

Increased funding for Medicaid would significantly decrease differential payment rates for different patient populations. Underfinancing of Medicaid causes notoriously low payment rates, which has been shown to worsen health disparities for beneficiaries by limiting their access to care.^28^ Payment reforms, however well‐intentioned and well‐designed, cannot change existing inequality in funding for providers who serve a disproportionate number of patients with Medicaid unless accompanied by financing reform. Medicare's disproportionate share hospital (DSH) payments, intended to direct greater funding to facilities serving higher percentages of Medicaid and/or uninsured patients, set a national precedent for strategies that address historical inequity in providers' resources.

Another tool that has the potential to address this root cause of inequity is all‐payer rate setting, which entails the regulation of prices that hospitals and/or other providers charge insurers and patients. Implemented by seven states in the 1970s, all‐payer rate setting fell out of favor during the 1980s as policy shifted from an emphasis on direct price regulation to managed care as a cost management tool. However, Maryland has continued rate setting and has yielded many lessons about the opportunities and challenges of such a system. Unlike most states—where large health systems hold significant price negotiating power with commercial insurers that want to keep that system in their network—Maryland does not allow hospitals to negotiate prices with insurers. Instead, the independent Health Services Cost Review Commission (HSCRC) directly controls payment rates for every hospital in the state.

One lesson from Maryland is the danger of implementing all‐payer rate setting in the context of fee‐for‐service payment structures; the state experienced significant total cost growth in the early 2000s as hospitals increased utilization in response to per‐service price controls.^29^ To counter this growth, the state additionally implemented hospital global budgets, which HSCRC uses to set the total revenue that a hospital will receive from all payers annually. Hospital global budgets, in conjunction with all‐payer rate setting, have been more successful in controlling total costs.^30^

A third tool to mitigate unequal distribution of resources between providers serving different populations is lump sum, up‐front payments to low‐revenue providers. CMMI has tested this concept—first with the Accountable Care Organization Investment Model (AIM), which provided up‐front infrastructure payments to rural Accountable Care Organizations, then with adjustments to benchmarks and other payment rates (e.g., social risk adjustment) using the Area Deprivation Index (ADI) scores of providers' patient populations or patient self‐reported data. There is much additional research to be done in this area, including exploring whether ADI or other methods is the best way to identify providers in need of additional funding and whether particular metrics, conditions of funding, or monitoring strategies are most effective in ensuring that the increased funding leads to greater equity.

AHRQ's research expertise would be helpful in assessing different tools for redistributing funding between providers serving different patient populations, particularly in evaluating the effect that increasing Medicaid payment rates, Maryland's all‐payer model, and CMMI's experiments in providing additional payments to certain providers have on health equity.

### 4.3 Scarcity of financing

One of the most significant financing‐related barriers to health equity is inadequate financing for critical health‐related social services. It is well understood that factors such as stable housing, food security, and early childhood education are correlated with better health outcomes. However, public financing of social services compared with medical services is lower in the United States than in other Organization for Economic Cooperation and Development (OECD) nations.^31^ While there has been some recognition that addressing social drivers of health is necessary to improve health outcomes, many strategies for doing so have relied on healthcare providers to either create their own interventions or develop stronger partnerships with under‐resourced community‐based organizations (typically with limited or no additional payment). Greater public and private financing of critical social services could promote health equity by improving social conditions necessary for better health.

Even some social services covered by health insurance are underfinanced. One prominent example is home‐and‐community‐based services (HCBS) that are covered through Medicaid—one of the most poorly financed sources of health coverage. Either greater Medicaid financing of HCBS and/or requiring Medicare and commercial insurers to also contribute to HCBS financing could allow more people who are eligible for those services to access them.^32^ For example, all insurers could be required to cover some level of HCBS as an essential benefit.

Primary care is another prominent example of an underfinanced healthcare service. The National Academies of Sciences, Engineering, and Medicine noted in its 2021 report “Implementing High‐Quality Primary Care: Rebuilding the Foundation of Health Care” that primary care is both chronically underfunded and essential to the provision of equitable, whole‐person care.^33^ The report recommended greater funding for primary care overall, as well as the use of clinical and social risk adjustment to ensure that primary care teams serving the most vulnerable patients have the resources to address their needs.

The domain of scarce financing offers AHRQ opportunities for both research and convening. While there is an abundance of evidence regarding the prevalence of social determinants of health, there is a relative lack of research on the best financing interventions to address them. More research is also needed on innovative financing mechanisms for HCBS—including moving financing outside of Medicaid or even outside of health insurance altogether.

Federal agencies, private payers, and state policymakers are interested in addressing social determinants of health but lack evidence‐based financing strategies for doing so. AHRQ could play a role in convening leaders in health, housing, and education to better understand the state of the evidence and promising paths to better financing.

### 4.4 Lack of service integration and clear accountability for outcomes

Financing for all social services does not—and need not—flow through health care. Housing, education, and other social supports would be worth financing adequately regardless of their effect on health outcomes. However, since no one entity is held accountable for failures to address whole‐person health, no one entity has an incentive to ensure that patients have access to all the services they need.

There are several examples of attempts to better integrate financing for health and health‐related social needs. The Collaborative Approach to Public Good Investment (CAPGI) model, which creates a governance structure for multiple private and public entities to collaboratively finance interventions that address social determinants of health rather than assigning all financing and accountability to a single sector, is currently being tested in multiple pilot sites.^34^ States have also begun using 1115 waivers to test Medicaid financing of social services: California is using “in‐lieu‐of” services to provide housing through Medicaid, Oregon, is providing a variety of social services (including housing, food assistance, and employment assistance) to Medicaid beneficiaries experiencing transitional events such as homelessness or incarceration, and North Carolina is using Medicaid funding to finance Healthy Opportunity Pilots that test interventions to address social drivers of health.

AHRQ could contribute to this area by evaluating strategies tested to‐date and working with partners to develop new, innovative ways to integrate the financing and administration of social services influencing health.

### 4.5 Patient cost barriers to care

Insurers have attempted some experiments in adjusting patient cost‐sharing to encourage better access. Notably, value‐based insurance design (VBID) is a model in which insurers reduce cost‐sharing for targeted, high‐value services (such as chronic disease management services for patients with diabetes).^35^ AHRQ could contribute to research into whether VBID or similar reforms improve access and outcomes for specific populations, such as people with lower incomes. AHRQ could also contribute to research to refine the definition of high‐value versus low‐value care, especially for services and procedures that differ in value depending on the context.

Despite voluntary efforts of insurers, payment and financing reforms to address patient cost barriers to care may be difficult to achieve without significant changes to the structure of US healthcare. Absent Medicaid expansion or other legislative reforms to give more US residents access to publicly funded health insurance with low or no cost‐sharing, there are limited options for addressing patient cost as a barrier to care and a source of inequity.

### 4.6 Bias in provider behavior and diagnostic tools

There is ample evidence of bias in both provider behavior and diagnostic tools, including the examples cited in Table 1. This domain is one where an existing payment reform—pay‐for‐performance—could be modified to incentivize better care for patients whose care is worsened by bias (including non‐white people, people with disabilities, and other populations). A systematic literature review of performance‐based payment models found that they have the potential to either reduce or widen disparities, depending on design.^36^

The development of appropriate and effective equity‐related measures would be a prime opportunity for AHRQ engagement. In particular, AHRQ could ensure that the patient voice is heard by helping identify ways to incorporate patient‐driven and/or patient‐reported outcome measures into pay‐for‐performance models.

### 4.7 Better data to support financing and payment reforms

Humility should accompany the design of any financing and payment reforms. Many external factors may interact with these reforms, such as those mentioned in Section 1.1 along with competitive market dynamics, workforce supply, and community culture. Whenever possible, policymakers and researchers should proactively identify and measure these factors, to support policy refinements and more appropriate research inferences.

Current financing and payment strategies also had to rely on limited data about a population's racial/ethnic composition, socioeconomic status, disability status, gender identity, or other correlates of health inequity. For future reforms to have meaningful impact on health equity, urgent and meaningful investments by both public and private sectors to standardize and incentivize the collection of such data would be required to support decision‐making.

In summary, we recommend a number of ways that AHRQ could engage with financing and payment reform to promote health equity, described in Table 2.

**TABLE 2:** Summary of recommendations for Agency for Healthcare Research and Quality.

Root cause domain	Recommendations
Differential distribution of resources between providers	Support research to evaluate the effect that the following payment and financing reforms have on health equity: increasing Medicaid payment rates, Maryland's all‐payer model, and CMMI's experiments in providing additional payments to certain providers.
Scarcity of financing	Support research on innovative financing mechanisms for HCBS—including moving financing outside of Medicaid or even outside of health insurance altogether. Convene leaders in health, housing, and education from federal and state agencies and private payers to better understand the state of the evidence for addressing social determinants of health and promising paths to better financing.
Lack of integration of services and accountability for outcomes	Evaluate strategies to integrate financing for health and social services tested to‐date, such as CAPGI and Medicaid 1115 waivers that fund housing and other social services. Work with partners to develop new, innovative ways to integrate the financing and administration of social services influencing health.
Patient cost barriers to care	Contribute to research into whether VBID or similar reforms improve access and outcomes for specific populations, such as people with lower incomes. Refine the definition of high‐value versus low‐value care, especially for services and procedures that differ in value depending on the context.
Bias in provider behavior and diagnostic tools	Identify ways to incorporate patient‐driven and/or patient‐reported outcome measures into pay‐for‐performance models that measure biased treatment.

## 5 CONCLUSION

While we lack rigorous evidence on what financing and payment strategies would be most transformative in advancing health equity, examining the root mechanisms of how current financing and payment policies exacerbate or reinforce inequity reveals myriad opportunities to test promising payment and financing models. AHRQ could assist in these efforts by bringing the strength of its research, convening, and technical expertise to advance knowledge and policy practice in this area.

## Supporting information


**Supporting Information S1.** Supporting Information.

## References

[1] Berenson R , Ginsburg PB , Christianson JB , Yee T . The growing power of some providers to win steep payment increases from insurers suggests policy remedies may be needed. Health Aff. 2012;31:973‐981. 10.1377/hlthaff.2011.0920 22566436

[2] Medicaid and CHIP Payment and Access Commission (MACPAC) . Managed care's effect on outcomes. Accessed June 22, 2023. https://www.macpac.gov/subtopic/managed-cares-effect-on-outcomes/

[3] Centers for Medicare & Medicaid Services . Innovation center strategy refresh. 2021. Accessed June 22, 2023. https://innovation.cms.gov/strategic-direction-whitepaper

[4] Health Care Payment Learning & Action Network . APM measurement: progress of alternative payment models, 2022 methodology and results report. 2022. Accessed June 22, 2023. http://hcp-lan.org/workproducts/APM-Methodology-2022.pdf

[5] Edmiston KD . Alternative Payment Models, Value‐Based Payments, and Health Disparities. Center for Insurance Policy and Research; 2022.

[6] Yasaitis L , Pajerowski W , Polsky D , Werner RM . Physicians' participation in ACOs is lower in places with vulnerable populations than in more affluent communities. Health Aff. 2016;35(8):1382‐1390. 10.1377/hlthaff.2015.1635 PMC5053882 27503961

[7] Liao J , Ibrahim SA , Huang Q , et al. The proportion of marginalized individuals in US communities and hospital participation in bundled payments. Popul Health Manag. 2022;25:501‐508. 35532549 10.1089/pop.2021.0334 PMC9419980

[8] Lin SC , Joynt Maddox KE , Ryan AM , Moloci N , Shay A , Hollingsworth JM . Exit rates of accountable care organizations that serve high proportions of beneficiaries of racial and ethnic minority groups. JAMA Health Forum. 2022;3(9):e223398. 36218951 10.1001/jamahealthforum.2022.3398 PMC9526083

[9] Eberth JM , Hung P , Benavidez GA , et al. The problem of the color line: spatial access to hospital services for minoritized racial and ethnic groups. Health Aff. 2022;41:237‐246. 10.1377/hlthaff.2021.01409 35130071

[10] Cooper Z , Stiegman O , Ndumele CD , Staiger B , Skinner J . Geographical variation in health spending across the US among privately insured individuals and enrollees in Medicaid and Medicare. JAMA. 2022;5(7):e2222138. 10.1001/jamanetworkopen.2022.22138 PMC9301520 35857326

[11] Commonwealth Fund . Medicaid's value. 2019. Accessed June 22, 2023. 10.26099/m821-mp71

[12] CMS . National health expenditures data. 2021. Accessed June 22, 2023. https://www.cms.gov/research-statistics-data-and-systems/statisticstrends-and-reports/nationalhealthexpenddata

[13] Glaser LB . Quality of Medicaid varies as a result of public policy. *Cornell Chronicle*. 2018.

[14] Kaiser Family Foundation . Medicaid HCBS waiver waiting list enrollment, by target population and whether states screen for eligibility. 2021. Accessed June 22, 2023. https://www.kff.org/medicaid/state-indicator/medicaid-hcbs-waiver-waiting-list-enrollment-by-target-population-and-whether-states-screen-for-eligibility/?currentTimeframe=0&sortModel=%7B%22colId%22:%22Location%22,%22sort%22:%22asc%22%7D

[15] Gorges RJ , Sanghavi P , Konetzka RT . A national examination of long‐term care setting, outcomes, and disparities among elderly dual eligibles. Health Aff. 2019;38:1110‐1118. 10.1377/hlthaff.2018.05409 PMC7147241 31260370

[16] Medicaid and CHIP Payment and Access Commission . People with disabilities. 2022. Accessed June 22, 2023. https://www.macpac.gov/subtopic/people-with-disabilities/

[17] U.S. Department of Education . About the individuals with disabilities education act. 2022. Accessed June 22, 2023. https://sites.ed.gov/idea/about-idea/

[18] Finkelstein A . Why it's so hard to cut waste in health care. *The New York Times*. 2021.

[19] Hall WJ , Chapman MV , Lee KM , et al. Implicit racial/ethnic bias among health care professionals and its influence on health care outcomes: a systematic review. Am J Public Health. 2015;105:2588. 10.2105/AJPH.2015.302903 PMC4638275 26469668

[20] Institute of Medicine (US) Committee on Understanding and Eliminating Racial and Ethnic Disparities in Health Care . Unequal Treatment: Confronting Racial and Ethnic Disparities in Health Care. National Academies Press; 2003. 25032386

[21] Tong M , Artiga S . Use of Race in Clinical Diagnosis and Decision Making: Overview and Implications. Kaiser Family Foundation; 2021.

[22] Garfield R , Damico A . The Coverage Gap: Uninsured Poor Adults in States that Do Not Expand Medicaid. Kaiser Family Foundation Issue Brief; 2021.

[23] Hoffman KM , Trawalter S , Axt JR , Oliver MN . Racial bias in pain assessment and treatment recommendations, and false beliefs about biological differences between blacks and whites. Proc Natl Acad Sci U S A. 2016;113:4296‐4301. 27044069 10.1073/pnas.1516047113 PMC4843483

[24] Alkhouli M , Alqahtani F , Holmes DR , Berzingi C . Racial disparities in the utilization and outcomes of structural heart disease interventions in the United States. J Am Heart Assoc. 2019;8:e012125. 31315490 10.1161/JAHA.119.012125 PMC6761641

[25] Bradley EA , Hollins S . Assessment of patients with intellectual disabilities. In: Goldbloom DS, eds. Psychiatric Clinical Skills, Indian Journal of Psychiatry. Elsevier; 2006:194‐210.

[26] Sjoding MW , Dickson RP , Iwashyna TJ , Gay SE , Valley TS . Letter to the editor: racial bias in pulse oximetry measurement. N Engl J Med. 2020;383:2477‐2478. 33326721 10.1056/NEJMc2029240 PMC7808260

[27] Allen H , Golberstein E , Bailey Z . Eliminating health disparities will require looking at how much and how Medicaid pays participating providers. Milbank Q. 2022;February 2022.

[28] Hsu C‐y , Yang W , Parikh RV , et al. Race, genetic ancestry, and estimating kidney function in CKD. N Engl J Med. 2021;385:1750‐1760. 34554660 10.1056/NEJMoa2103753 PMC8994696

[29] Murray R , Berenson RA . Hospital Rate Setting Revisited. Urban Institute; 2015.

[30] Butler S , Matthew DB , Cabello M . Re‐Balancing Medical and Social Spending to Promote Health: Increasing State Flexibility to Improve Health through Housing. Brookings Institute; 2017.

[31] Chidambaram P . Potential Impact of Additional Federal Funds for Medicaid HCBS for Seniors and People with Disabilities. Kaiser Family Foundation; 2021.

[32] Emanuel EJ , Johnson DW , Guido M , Goozner M . Meaningful value‐based payment reform, part 1: Maryland leads the way. Health Aff. 2022.

[33] The National Academies of Sciences, Engineering, and Medicine . Implementing High‐Quality Primary Care: Rebuilding the Foundation of Health Care. National Academies Press (US); 2021. 34251766

[34] Chernew M , Rosen AB , Fendrick AM . Value‐based insurance design. Health Aff. 2007;26:192‐203. 10.1377/hlthaff.26.2.w195 17264100

[35] Conway A . The role of pay‐for‐performance in reducing healthcare disparities: a narrative literature review. Prev Med. 2022;164:107274. 36156282 10.1016/j.ypmed.2022.107274

[36] Altman D . Persistent Vaccine Myths. Kaiser Family Foundation; 2021. Accessed June 22, 2023. https://www.commonwealthfund.org/grants/collaborative‐approach‐public‐good‐investment‐capgi‐proposalenable‐more‐communities

